# A matrix representation for sustainable activities

**DOI:** 10.1038/s41598-022-05750-6

**Published:** 2022-02-09

**Authors:** A. S. Mahmud, D. S. Zachary

**Affiliations:** Krieger School of Arts and Sciences (Advanced Academic Programs), Johns Hopkins University, 1717 Massachusetts Ave., N.W., Washington, DC 20036 USA

**Keywords:** Energy infrastructure, Renewable energy

## Abstract

The present paper develops a matrix framework to determine the sustainability of multiple activities simultaneously. We define an activity as an action or process involving multiple resources; each activity depends upon other activities at a higher level. First, the problem of sustainability is framed in the context of an input–output model. Later, an infinite layer approach is adopted to represent different sectors of the economy and both renewable and non-renewable resources. Using the infinite layer approach, the concentration of renewable energy usage is calculated for each activity. The conversion to productive use varies from one resource to another. The current endeavor also focuses on enhancing energy efficiency to reduce non-renewable resource dependency.

## Introduction

Multiple activities require various primary energy resources in an economy. Moreover, different activities of the production economy interact with each other at different levels. For example, to produce industrial goods, one needs energy resources. To transport the raw materials of the final industrial output, the firms need transportation facilities. The transportation sector itself consumes energy. Therefore, to address the question of sustainability, we need to consider all activities in multiple sectors along with all the energy resources.

Given the above background, specific energy policy questions arise: What is the best renewable energy to invest in given the efficiency of all systems, technologies, and contributing processing used to deliver that energy? The complement of this question is: What non-renewable energy sources should be prioritized for reducing emissions?

The present paper develops a matrix framework to determine the sustainability of multiple activities simultaneously. We define an activity as an action or process involving multiple resources. First, the problem of sustainability is framed in the context of an input–output model. In the input–output model, all activities depend upon primary energy resources that include both renewable and non-renewable. Furthermore, each activity depends upon other activities at a higher level. Therefore, we can calculate the aggregate contribution of each activity in the consumption of energy in the economy.

In the following section, an infinite layer approach following Zachary^1^ is adopted to represent different sectors of the economy and both renewable and non-renewable resources. The first layer includes energy that is directly used in each activity. The second layer of energy is utilized to produce the first layer of production. So, on and so forth. Again, the infinite layer approach allows calculating the concentration of renewable energy usage for each activity. The breakdown of sectors and infinite layer approach enable us to calculate the aggregate energy consumption in multiple sectors in the economy at the same time simultaneously. We illustrate a specific example where four different but interconnected sectors of the economy are represented: industrial, residential, commercial, and transportation.

The current endeavor also focuses on enhancing energy efficiency to reduce non-renewable resource dependency. In the last model, we discuss the efficacy of investment in increasing the efficiency of both renewable and energy resources. The important finding is that a more effective policy from the sustainability point could increase the efficiency of a non-renewable energy source^2^. At the aggregate level, the paper identifies the concentration of renewable energy usage across different sectors. A policy combination of taxes and subsidies in the economy as a whole can be drawn using this information. Also, conversion to productive use is not uniform. The present context also discusses how an economy can maximize the return on research and development investment research and development investment return to improve efficiency. In summary, we develop the general sustainability problem from Zachary^1^ in a matrix framework focusing upon multiple and simultaneous activities in contrast to a single activity.

The hidden energy cost for renewable energy systems (RES) is sometimes not fully taken into account when the issue of considered when sustainability is discussed^3^. Authors de Groot and le Pair point out that “wind-generated electricity requires back up the capacity of conventional power stations...when wind supply is falling short.” More generally, understanding the economic challenges of mixed renewable and fossil energy systems requires a full understanding of all supporting energy systems. A model to understand all supporting systems was discussed by Zachary. The sustainability condition was determined for a given duration and cost. This condition was determined if and only if all the proceeding activities and their respective energy and resources satisfy the notion of sustainability.

In contrast to Zachary, where only activity was considered, the current endeavor is concerned with multiple activities at the aggregate level. Since energy sources are required for all activities while activities themselves are related to production relations, a matrix framework is developed to address the problem. Production relations across multiple activities can be expressed in an input–output matrix. Furthermore, different layers of production can also be manifested in the adopted framework.

Renewable energy replacement considerations are also be considered in view of other attributes, including the popular importance-performance analysis by Chen et al.^4^. This tool considered actions both in terms of importance and performance^5^ and has been used in a number of applications including competitive analysis (e.g. Pezeshki and Mousavi^6^) and employment impacts (Garrett-Peltier^7^). The latter, for example, utilizes matrices to analyze the economic and environmental impacts of renewable energy and describes the employment generation when the economy is transforming towards cleaner energy.

Cost and the related technical considerations must also be considered in terms of replacement^8^ as well as the closely-related added value consideration by Bagheri et al.^9^. Bagheri et al. also illustrate the path of green growth in the context of Canada that prioritizes economic and job opportunities without sacrificing environmental concerns.

The present endeavor does not focus upon economic impacts per se; our main aim is to analyze the sustainability of multiple activities in the economy.

A related question to ask in the present context is where money should be invested? Is it better to invest in renewable energy or use carbon sequestration? This question, posed by Turner in the 1990s^10^, represents the type of choices going forward to produce a cleaner energy portfolio. Our paper also discussed the potential avenues for R & D investment to increase the energy efficiency of the overall economy where the sustainability of multiple activities is concerned.

Understanding national politics of energy transition is key to having solutions available early enough to minimize the cost of impact. Work by Jacobsson and Lauber^11^ emphasizes this issue with the diffusion of renewable energy in Germany’s political system. Other authors emphasize the importance of niche markets. Kemp et al., for example, mentioned examples from history (e.g., the steam engine developed by Newcomen to pump water from mines). These driving forces represent only part of the story behind the dynamics of new markets. The relatively new market of renewable energy is driven by these forces, but also, in part, by economic, social, and environmental drivers^12^.

The matrix model is developed in “Model” section. ’Infinite layer approximation’ is developed in “Infinite layer approximation” section. A specific numerical application is shown in “Specific approximation” section. “Aggregate values” section calculates aggregate values. An application of the model is for innovation is demonstrated in “Efficiency” section. Finally, we provide a discussion and conclusion in “Discussion and conclusion” section.

## Model

Consider an economy producing a vector of activities ${\mathbf {a}}$ using activities themselves and resources (both renewable and non-renewable types) as inputs. The economy can be expressed as follows:

1 $$\begin{aligned} \left[ {\mathbf {a}}\right]= & {} \left[ {\mathbf {C}}\right] \mathbf {a+x} \end{aligned}$$

2 $$\begin{aligned} \left[ {\mathbf {a}}\right]= & {} \left[ \mathbf {I-C}\right] ^{-1}\left[ {\mathbf {x}} \right] \end{aligned}$$

3 $$\begin{aligned} \left[ {\mathbf {a}}\right]= & {} \left[ {\mathbf {M}}\right] \left[ {\mathbf {x}}\right] \end{aligned}$$

$\mathbf {C}$ is the input–output matrix or production relations among activities. Without any source of energy sources, none of this production can take place. Therefore, if the vector $\left[ {\mathbf {x}}\right]$ is null, no production can take place. $\left[ {\mathbf {x}}\right]$ is the amount of energy that is required to sustain a certain level of activity $\left[ {\mathbf {a}}\right]$.

The value, $\left[ {\mathbf {M}}\right]$ is also known as the inverted Leontief matrix. The matrix aggregates all the chains through which each activity contributes to the other. Expanding the matrix, we obtain:

$$\begin{aligned} \left[ \begin{array}{c} a_{1} \\ a_{2} \\ a_{3} \\ a_{4} \\ a_{5} \\ a_{6} \end{array} \right] =\left[ \begin{array}{cccccc} m_{11} &{} m_{12} &{} m_{13} &{} m_{14} &{} m_{15} &{} m_{16} \\ &{} &{} &{} &{} &{} \\ &{} &{} &{} &{} &{} \\ &{} &{} m_{43} &{} &{} &{} \\ &{} &{} &{} &{} &{} \\ &{} &{} &{} &{} &{} m_{66} \end{array} \right] \left[ \begin{array}{c} x_{1} \\ x_{2} \\ x_{3} \\ x_{4} \\ x_{5} \\ x_{6} \end{array} \right] \end{aligned}$$

The total production of good $a_{i}$ is:

4 $$\begin{aligned} a_{i}= & {} \sum _{i^{\prime }=1}m_{ii^{\prime }}x_{i^{\prime }}. \end{aligned}$$

5 $$\begin{aligned} A= & {} \sum _{i=1}a_{i}=\sum _{i=1}\sum _{i^{\prime }=1}m_{ii^{\prime }}x_{i^{\prime }}. \end{aligned}$$

Production of each good (activity) $a_{i}$ requires resource the amount $x_{i}$. The required resource is obtained from both renewable and non-renewable sources. We assume that a certain portion $\alpha _{i}$ is produced from renewable and the rest from non-renewable sources:

6 $$\begin{aligned} x_{i}=x_{r, i}+x_{nr, i}; x_{r, i}=\alpha _{i}x_{i}, x_{nr, i}=\left( 1-\alpha _{i}\right) x_{i}. \end{aligned}$$

The total energy required is as follows:

7 $$\begin{aligned} \sum _{i=1}x_{i}=x. \end{aligned}$$

The total production from renewable and non-renewable sources of energy are:

8 $$\begin{aligned} A_{r}= & {} \sum _{i=1}\sum _{i^{\prime }=1}m_{ii^{\prime }}\alpha _{i^{\prime }}x_{i^{\prime }}. \end{aligned}$$

9 $$\begin{aligned} A_{nr}= & {} \sum _{i=1}\sum _{i^{\prime }=1}m_{ii^{\prime }}(1-\alpha _{i^{\prime }})x_{i^{\prime }}. \end{aligned}$$

### Renewable energy applications

Since we want to produce more from a renewable energy, we raise the question which resource adjustment will have the great impact? By increasing $\alpha _{i^{\prime }}$ on $A_{r}$, we find:

10 $$\begin{aligned} \frac{\partial A_{r}}{\partial \alpha _{i^{\prime }}}=\sum _{i=1}m_{ii^{ \prime }}x_{i^{\prime }}. \end{aligned}$$

The total amount of energy required to produce this amount of goods can be found by summing over the activities. The impact of resource $x_{i^{\prime }}$ in the total production is as follows:

11 $$\begin{aligned} a^{i^{\prime }}= & {} \sum _{i=1}a_{ii^{\prime }}=x_{i^{\prime }}\sum _{i=1}m_{ii^{\prime }} \Rightarrow x_{i^{\prime }}=\frac{ \sum _{i=1}a_{ii^{\prime }}}{\sum _{i=1}m_{ii^{\prime }}}. \end{aligned}$$

12 $$\begin{aligned} x= & {} \sum _{i^{\prime }=1}x_{i^{\prime }}=\sum _{i^{\prime }=1}\left[ \frac{ \sum _{i=1}a_{ii^{\prime }}}{\sum _{i=1}m_{ii^{\prime }}}\right] . \end{aligned}$$

where $a_{ii^{\prime }}$ is the contribution of good $i^{\prime }$ in the production of good *i* and $a^{i^{\prime }}$ is the total contribution from good $i^{\prime }$ in the entire economy. Whereas $a_{i^{\prime }}$ is the total production of good $i^{\prime }$; hence., $a^{i^{\prime }}$ is different from $a_{i^{\prime }}$. Suppose $i^{\prime }$ is the agriculture sector. Many other sectors besides agriculture itself including manufacturing, transportation depend upon agriculture directly and indirectly. $a^{i^{\prime }}$ encapsulates the contribution of agriculture to all such sectors and $a_{i^{\prime }}$ is the entire agricultural production The resource used for the sole purpose of producing good $i^{\prime }$ is $x_{i^{\prime }}$. The total resource utilized in the economy is *x*.

### Expansion of $\left[ {\mathbf {M}}\right]$

The input–output model described the activities of the present period as a function of activities of the last period and the energy used in the current period:

13 $$\begin{aligned} \left[ {\mathbf {a}}(t-1)\right] +\left[ {\mathbf {x}}\left( t\right) \right] \rightarrow \left[ {\mathbf {a}}\left( t\right) \right] \end{aligned}$$

If we expand the matrix $\left[ {\mathbf {M}}\right]$ into an infinite series, we obtain:

14 $$\begin{aligned} \left[ {\mathbf {a}}\right]= & {} \left[ {\mathbf {M}}\right] \left[ {\mathbf {x}}\right] \end{aligned}$$

15 $$\begin{aligned}= & {} \left[ \mathbf {I-C}\right] ^{-1}\left[ {\mathbf {x}}\right] \end{aligned}$$

16 $$\begin{aligned}= & {} \left[ {\mathbf {x}}\right] +\left[ {\mathbf {C}}\right] \left[ {\mathbf {x}} \right] +\left[ {\mathbf {C}}^{{\mathbf {2}}}\right] \left[ {\mathbf {x}}\right] +\cdots \end{aligned}$$

The first term in the series is the direct usage in the current period, *t*. The second term is the energy used during the last period, $(t-1)$ for activities to be utilized as inputs in the current period. The activities of the period $(t-1)$ itself depend upon activities of the period $(t-2)$ as inputs. The third term describes the energy content of the period $(t-2)$ that is involved in the production of the current period, *t*. By showing how the past activities relate to the present, the matrix expansion illustrates the total energy content to sustain a certain level of activities.

## Infinite layer approximation

We alternatively view production using a an infinite layer approach. This approach models the capacity levels of Zachary^1^. The first layer is energy that is directly used in each activity. The second layer is the energy that is used to produce the means of production that produces the first layer. The third layer describes the energy that is utilized to produce the second layer and so on and so forth. The first layer is customized to according to each activity specifically. However, supporting level activities require contributions of still other supporting activities.

In terms of energy, each successive layer subsequently contributes less energy content to support the immediate activity in question. Supporting levels contributed to other activities and therefore dilute their energy contribution. Using the example from Zachary^1^, the truck carrying apples for the lunch for the bicycler is also used for many other activities. The trucks energy is therefore diluted and only a small fraction is used in the bicycle trip activity.

In the previous section, all energy sources were added into one column; here, each energy source is now considered separately. About three non-renewable and six renewable energy sources are taken into account. Therefore, $\left[ {\mathbf {x}}\right]$ is no longer a vector but a matrix itself.

A simple analogy from Roemer^13^ could further assist our understanding of the techno-economy model. Consider an economy that produces corn using only labor and corn as input. Each unit of corn requires $c<1$ corn and 1 unit of labor. To maintain the current level of production of corn, the society requires *cL* units of corn and *L* units of labor. Therefore, the first layer of production requires *L* units of labor. The second layer requires *cL* units of labor to produce the required unit of corn as seed. The third layer requires $c^{2}L$ units of labor. Therefore, the aggregate requirement becomes:

$$\begin{aligned} L+cL+c^{2}L+\cdots \end{aligned}$$

Now we frame the arbitrary matrix [**M**] (Eq.14) in terms of techno-economy subsectors [${\mathbf {T}}$] and consider the set of energy sources that are readily available, both renewable and non-renewable energy sources. Recalling, an activity is described using a list of resources, (previously, $x^{1}:=\{{\mathrm{'tuna', 'salad', 'bread',}} \mathrm{'milk'}\}$, $n=4$). Now, in terms of technology sub-sectors, we define $x^{1}:=\{{ x^1_I,~x^1_T,~x^1_C,~x^1_R}\}$, representing industry, transportation, commercial, and residential sub-sectors. We expect the initial (top-level) activity to be customized according to the specific situation but the next layer, represented by [**T**] is generic and depends on the available energy situation. Furthermore, each supporting layer would have similar dependencies. There are the energy flows required to support the activity, or more specifically, produced the technology used in the supporting level. The contribution of the nth level is therefore,

17 $$\begin{aligned} i(n)=\beta (n)\cdot T. \end{aligned}$$

**Figure 1:**
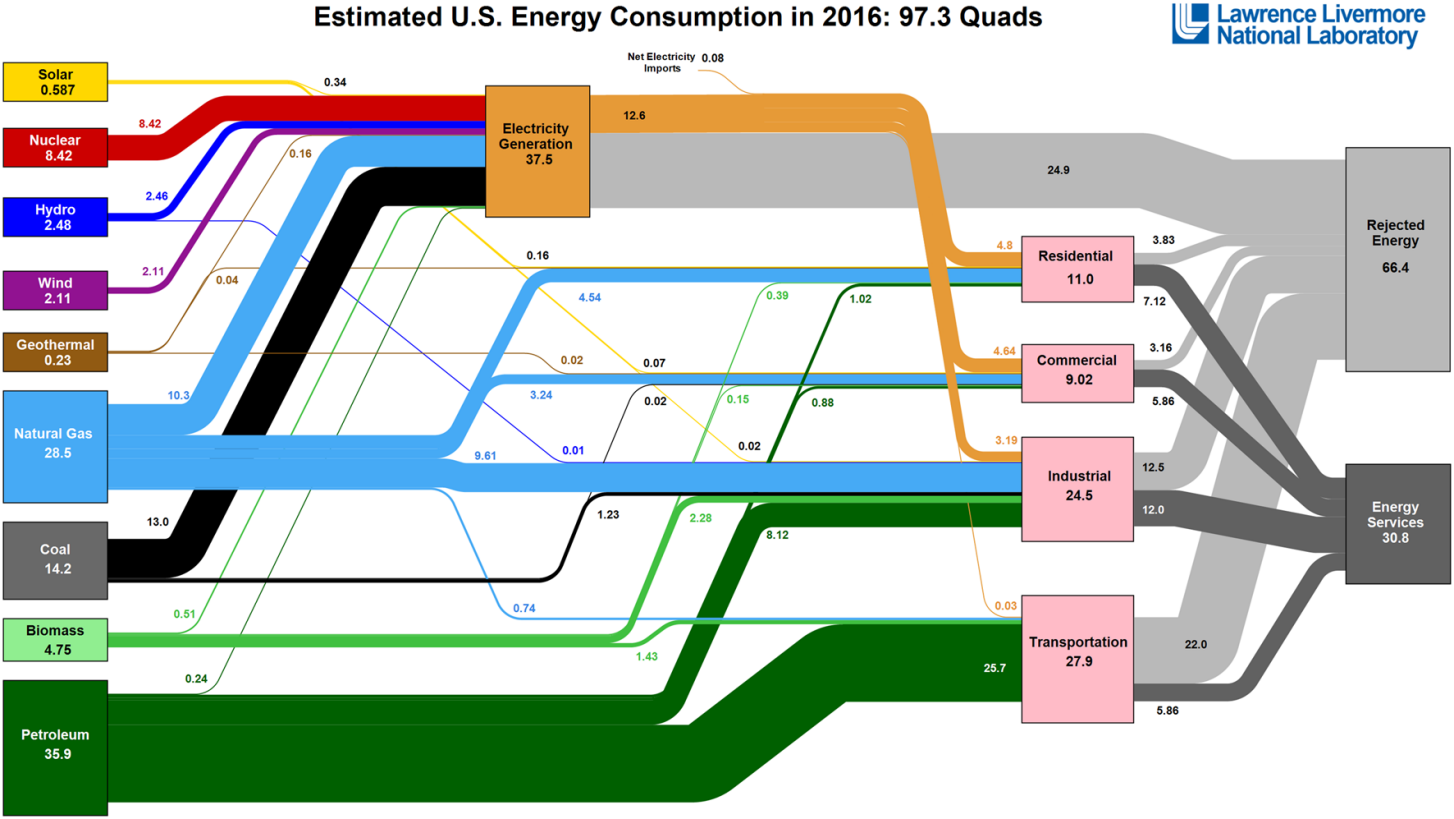
2016 Energy flow Chart for the USA. This figure is not covered by the CC BY license. (Lawrence Livermore National Labs)^14^. All rights reserved, used with permission.

The term $\beta (n)$ is a monotonically decreasing function, representing the fraction of energy content for higher supporting levels (Fig. 1,^1^). This is a technology matrix $[{\mathbf {T}}]$:

18 $$\begin{aligned} T=\left[ \begin{array}{cccc} t_{11} &{} t_{21} &{} t_{31} &{} t_{41} \\ t_{12} &{} t_{22} &{} t_{32} &{} t_{42} \\ t_{13} &{} t_{23} &{} t_{33} &{} t_{43} \\ t_{14} &{} t_{24} &{} t_{34} &{} t_{44} \end{array} \right] . \end{aligned}$$

The $[{\mathbf {T}}]$ matrix has constant elements for any *n* and represents a $4 \times 4$ matrix that contains the input–output values, similar to the construction of [**M**]. The inputs and outputs of the fifth sector, the electric sector, are intermediate energy flows and not represented in [**T**], but none-the-less will be considered the calculation of each energy source. The initial resources (first level) is represented as:

19 $$\begin{aligned} x^{1}=\left[ \begin{array}{c} x_{1,1}^{1}\cdots x_{1,9}^{1} \\ x_{2,1}^{1}\cdots x_{2,9}^{1} \\ x_{3,1}^{1}\cdots x_{3,9}^{1} \\ x_{4,1}^{1}\cdots x_{4,9}^{1} \end{array} \right] , \end{aligned}$$

and therefore the general energy expression (activity) is,

20 $$\begin{aligned}{}[{\mathbf {a}}]=x^{1}~+~\sum _{n}\beta (n)T_{i,j}\cdot x_{i,j}. \end{aligned}$$

The values $i=1,\ldots 4$ represent the technology sub-sectors: Industry, Transportation, Commercial, and Residential (See Fig. 1). The values $\upsilon =1,\ldots 9$ represent the six renewable energy sources: solar, hydro, wind, geothermal, biofuels, and nuclear energy, and the three non-renewable (fossil fuel) energies, natural gas, coal, and petroleum. Nuclear energy can be argued as renewable energy. Specific values of [**T**] are identified using estimates from the U.S. Department of Energy.

### Specific approximation

Empirical data shows that the energy required for an activity is large compared to the energy required to to produce the technology needed for that activity. An example is helpful: it takes approximately 260 gallons of petroleum to produce a car weighing 3000 pounds. Given a 15-year lifetime for a car and assuming a consumption of 750 gallons per year (corresponding to an average of 15,000 miles for 1 year and consuming on average 20 miles per gallon) the consumption over the 15 year lifetime is 11,250 gallons, giving a ratio of $\beta _{nr}\approx 1/43$^15^. A renewable energy system, producing energy in this case as compared to using it in the above car example, also requires energy to produce the technology. This amount of energy is also small compared to the energy produced by the turbine during its lifetime, $\beta _{r}\approx 1/20$ as a conservative value (Kubiszewski et al. 2010). The converging terms are $\sum _{n=1}^{\infty }(\beta _{r})^{n}=1/(1-\beta _{r})-1$, or specifically, $\sum _{n=1}^{\infty }(1/20)^{n}=0.053$ for renewable energies and $\sum _{n=1}^{\infty }(\beta _{nr})^{n}=0.024$. The actual value of $\beta$ probably is slightly larger than $\beta _{nr}$ since the techno-economy is comprised primarily of non-renewable technologies. Necessarily, the first level energy resource is equivalent to the *n*th level resource in this approximation,

21 $$\begin{aligned} x^{1}=x. \end{aligned}$$

Assuming a constant value for $\beta _{r}$ and $\beta _{nr}$ for renewable and non-renewables,

22 $$\begin{aligned}{}[{\mathbf {a}}_{r}]= & {} x_{r}^{1}~+~\frac{\beta _{r}}{1-\beta _{r}} T_{i,j}\cdot x_{r,i,j}, \end{aligned}$$

23 $$\begin{aligned}{}[{\mathbf {a}}_{nr}]= & {} x_{nr}^{1}~+~\frac{\beta _{nr}}{1-\beta _{nr}} T_{i,j}\cdot x_{nr,i,j}. \end{aligned}$$

For the six renewable energy sources. As a zero-emissions fuel, we group nuclear with renewable energy. The first level values for renewable energies are,

24 $$\begin{aligned} x_{r}^{1}=\left[ \begin{array}{c} x_{r,1,1}^{1}\ldots x_{r,1,6}^{1} \\ x_{r,2,1}^{1}\ldots x_{r,2,6}^{1} \\ x_{r,3,1}^{1}\ldots x_{r,3,6}^{1} \\ x_{r,4,1}^{1}\ldots x_{r,4,6}^{1} \end{array} \right] \end{aligned}$$

and for non-renewable energies they are,

25 $$\begin{aligned} x_{nr}^{1}=\left[ \begin{array}{c} x_{nr,1,7}^{1}\ldots x_{nr,1,9}^{1} \\ x_{nr,2,7}^{1}\ldots x_{nr,2,9}^{1} \\ x_{nr,3,7}^{1}\ldots x_{nr,3,9}^{1} \\ x_{nr,4,7}^{1}\ldots x_{nr,4,9}^{1} \end{array} \right] \end{aligned}$$

We drop the superscript on the activity on the r.h.s. as this now represents a general activity. Combining above equations, we obtain:

26 $$\begin{aligned}{}[{\mathbf {a}}_{r}]=\left[ \begin{array}{c} x_{r,1,1}^{1}\ldots x_{r,1,6}^{1} \\ x_{r,2,1}^{1}\ldots x_{r,2,6}^{1} \\ x_{r,3,1}^{1}\ldots x_{r,3,6}^{1} \\ x_{r,4,1}^{1}\ldots x_{r,4,6}^{1} \end{array} \right] ~+~\frac{\beta _{r}}{1-\beta _{r}}\left[ \begin{array}{cccc} t_{11} &{} t_{21} &{} t_{31} &{} t_{41} \\ t_{12} &{} t_{22} &{} t_{32} &{} t_{42} \\ t_{13} &{} t_{23} &{} t_{33} &{} t_{43} \\ t_{14} &{} t_{24} &{} t_{34} &{} t_{44} \end{array} \right] \cdot \left[ \begin{array}{c} x_{r,1,1}\ldots x_{r,1,6} \\ x_{r,2,1}\ldots x_{r,2,6} \\ x_{r,3,1}\ldots x_{r,3,6} \\ x_{r,4,1}\ldots x_{r,4,6} \end{array} \right] , \end{aligned}$$

and similarly for non-renewables

27 $$\begin{aligned}{}[{\mathbf {a}}_{nr}]=\left[ \begin{array}{c} x_{nr,1,7}^{1}\ldots x_{nr,1,9}^{1} \\ x_{nr,2,7}^{1}\ldots x_{nr,2,9}^{1} \\ x_{nr,3,7}^{1}\ldots x_{nr,3,9}^{1} \\ x_{nr,4,7}^{1}\ldots x_{nr,4,9}^{1} \end{array} \right] ~+~\frac{\beta _{nr}}{1-\beta _{nr}}\left[ \begin{array}{cccc} t_{11} &{} t_{21} &{} t_{31} &{} t_{41} \\ t_{12} &{} t_{22} &{} t_{32} &{} t_{42} \\ t_{13} &{} t_{23} &{} t_{33} &{} t_{43} \\ t_{14} &{} t_{24} &{} t_{34} &{} t_{44} \end{array} \right] \cdot \left[ \begin{array}{c} x_{nr,1,7}\ldots x_{nr,1,9} \\ x_{nr,2,7}\ldots x_{nr,2,9} \\ x_{nr,3,7}\ldots x_{nr,3,9} \\ x_{nr,4,7}\ldots x_{nr,4,9} \end{array} \right] , \end{aligned}$$

Energy policies can benefit by having detailed knowledge of the cost of energy change, not only at the top-level but also for all supporting levels. Details of energy processes of supporting process is difficult to assess resulting from the uncertainty of the contribution of supporting processes. Each supporting process that is further removed from the top-level has rapidly increasing uncertainty. The National Renewable Energy Laboratory (NREL) has none-the-less developed rules and cut-off LCA procedures^16^ to partially address this related issue.

In this work, an example of an energy transfer matrix to address an infinite number of supporting layers can be constructed for the four energy sectors would have elements that connect the other three sectors: [$${\mathbf {T}}$$]:$$ t_{11}$$ (industry to industry), $$t_{22}$$ (transportation to industry), $$ t_{33}$$ (commercial to commercial), $$t_{44}$$ (residential to residential). A weak coupling between sectors would result in $$T=1$$, a good first order approximation.

The total estimated energy for the USA is: 97.4 Quads (1 quad is equal to a 1015 BTUs, a short-scale quadrillion) or 1:055 1018 joules. According to the Energy Information Administration (EIA) the energy usage breaks down in the following way: Industry (22%), Transportation (29%)), Commercial (11%), and Residential (39%) (USA Energy Information Administration, Monthly Energy Review, April, 2017). The fraction of the energy to total energy demand are: natural gas (28.4%), coal (14.2%), petroleum (35.9 %), renewables (10.2%) and nuclear (8.4%), (USA primary energy consumption by section, 2017, USA Energy Information Administration, Monthly Energy Review, April, 2017). These are used in the various sectors.

### Aggregate values

An Aggregate energy value is obtained from by summing over technologies $i,\, {\widehat{a}}_{j}=\sum _{i}a\ _{r,i,j}$. Likewise, technology-based aggregates are found by energy sources $j,\ \ {\widetilde{a}}_{i}=\sum _{j}a\ _{r,i,j}$. The first-level technology aggregated values become,

28 $$\begin{aligned} {\widehat{x}}_{r}^{1}=\sum _{i}^{4}\left[ x_{r,1,1}^{1},\ldots ,x_{r,1,6}^{1}\right] \end{aligned}$$

and for non-renewables,

29 $$\begin{aligned} {\widehat{x}}_{nr}^{1}=\sum _{i}^{4}\left[ x_{nr,1,7},\ldots ,x_{nr,1,9}\right] \end{aligned}$$

Likewise, the energy-aggregated values become,

30 $$\begin{aligned} {\widetilde{x}}_{r}^{1}=\sum _{j=1}^{6}\left[ \begin{array}{c} x_{r,1,j}^{1} \\ x_{r,2,j}^{1} \\ x_{r,3,j}^{1} \\ x_{r,4,j}^{1} \end{array} \right] \end{aligned}$$

Earlier we defined $\alpha _{i}$ as the proportion of energy comes from renewable sources. The share of renewables in each sector is as follows:

31 $$\begin{aligned} \alpha _{i}=\frac{{\widetilde{x}}_{r}^{1}+\Omega _{r}\cdot {\mathbf {T}}\cdot {\widetilde{x}}_{r}}{{\widetilde{x}}_{r}^{1}+\Omega _{r}\cdot {\mathbf {T}}\cdot {\widetilde{x}}_{r}+{\widetilde{x}}_{nr}^{1}+\Omega _{nr}\cdot {\mathbf {T}}\cdot {\widetilde{x}}_{nr}} \end{aligned}$$

where

32 $$\begin{aligned} \Omega _{r}= & {} \frac{\beta _{r}}{1-\beta _{r}} \end{aligned}$$

33 $$\begin{aligned} \Omega _{nr}= & {} \frac{\beta _{nr}}{1-\beta _{nr}} \end{aligned}$$

The lowest $\alpha _{i}$ will identify the sector that has smallest portions of renewable resource usage. An increase in the production of the sector with the highest $\alpha _{i}$ will enhance the overall usage of renewable energy. In contrast, a decline in the production sector with the lowest $\alpha _{i}$ will decrease the overall dependence on non-renewables. By aggregating activities, we obtain:

34 $$\begin{aligned} {\widetilde{a}}={\widetilde{x}}_{r}^{1}+\frac{\beta _{r}}{1-\beta _{r}}{\mathbf {T}} \cdot {\widetilde{x}}_{r}+{\widetilde{x}}_{nr}^{1}+\frac{\beta _{nr}}{1-\beta _{nr}}{\mathbf {T}}\cdot {\widetilde{x}}_{nr} \end{aligned}$$

and the matrix values are,

35 $$\begin{aligned} \left[ \begin{array}{c} {\widetilde{a}}_{r1} \\ {\widetilde{a}}_{r2} \\ {\widetilde{a}}_{r3} \\ {\widetilde{a}}_{r4} \end{array} \right] =\left[ \begin{array}{c} \alpha _{1}{\widetilde{x}}_{1}^{1} \\ \alpha _{2}{\widetilde{x}}_{2}^{1} \\ \alpha _{3}{\widetilde{x}}_{3}^{1} \\ \alpha _{4}{\widetilde{x}}_{4}^{1} \end{array} \right] +\frac{\beta _{r}}{1-\beta _{r}}\left[ \begin{array}{cccc} t_{11} &{} t_{21} &{} t_{31} &{} t_{41} \\ t_{12} &{} t_{22} &{} t_{32} &{} t_{42} \\ t_{13} &{} t_{23} &{} t_{33} &{} t_{43} \\ t_{14} &{} t_{24} &{} t_{34} &{} t_{44} \end{array} \right] \cdot \left[ \begin{array}{c} \alpha _{1}{\widetilde{x}}_{1}^{1} \\ \alpha _{2}{\widetilde{x}}_{2}^{1} \\ \alpha _{3}{\widetilde{x}}_{3}^{1} \\ \alpha _{4}{\widetilde{x}}_{4}^{1} \end{array} \right] \end{aligned}$$

The derivative with respect to $\alpha _{i}$ is calculated in order to determine the increase or decrease of an energy use in a particular sector. For example,

36 $$\begin{aligned} {\widetilde{x}}_{i}^{1}+\frac{\beta _{r}}{1-\beta _{r}}\mathbf {\sum } _{i^{\prime }=1}t_{ii^{\prime }}{\widetilde{x}}_{i} \end{aligned}$$

This value calculates the immediate impact on the direct usage and the impact on the techno-economy as well. An increase in production of the sector with the highest $\alpha _{i}$ will increase the overall usage of renewable energy. In contrast, a decline in the production of the sector with the lowest $\alpha _{i}$ will decrease the overall usage of the non-renewable energy.

## Efficiency

We now explore the possibility of improving the efficiency of energy conversion. We can disaggregate the use of resources into two components: the raw energy and the level of efficiency in converting the raw energy. The first level disaggregation for renewable energies is as follows:

37 $$\begin{aligned} x_{r}^{1}=\left[ \begin{array}{cc} \epsilon _{r,1,1}^{1}{\widetilde{x}}_{r,1,1}^{1} & \epsilon _{r,1,6}^{1}{\widetilde{x}}_{r,1,6}^{1} \\ & \\ \epsilon _{r,4,1}^{1}{\widetilde{x}}_{r,4,1}^{1} &\epsilon _{r,4,6}^{1}{\widetilde{x}}_{r,4,6}^{1} \end{array} \right] \end{aligned}$$

where $\epsilon _{r,i,j}^{1}$ denotes efficiency and $\widetilde{x }_{r,i,j}^{1}$ the corresponding raw energy, and $\otimes$ indicates product by product multiplication between two matrices of equal dimensions. Similar disaggregation is done for higher levels:

38 $$\begin{aligned}{}[{\mathbf {a}}_{r}]={\widetilde{x}}_{r}^{1}\otimes \epsilon _{r}^{1}~+~ \frac{\beta _{r}}{1-\beta _{r}}T\cdot {\widetilde{x}}_{r}\otimes \epsilon _{r} \end{aligned}$$

and for non-renewables,

39 $$\begin{aligned}{}[{\mathbf {a}}_{nr}]={\widetilde{x}}_{nr}^{1}\otimes \epsilon _{nr}^{1}~+~ \frac{\beta _{nr}}{1-\beta _{nr}}T\cdot {\widetilde{x}}_{nr}\otimes \epsilon _{nr} \end{aligned}$$

We make the following simplifying assumptions:

**A 1:** The efficiency level of a particular energy in a specific technology is the same at all levels: $\epsilon _{r,i,j}^{1}=\epsilon _{r,i,j},\ \epsilon _{nr,i,j}^{1}=\epsilon _{nr,i,j}\ \forall \ i,\ j$.

**A 2:** The incremental change possible at a time is the same across all technologies:$(1-\delta )\left( 1-\epsilon _{r,i,j}\right)$.

To reduce the level of raw energy usage for non-renewables which can be achieved by either increasing the efficiency of renewables or non-renewables. We can increase ${\mathbf {a}}_{r}$ by increasing $\overline{ \epsilon }_{r,j}$ and decrease the use of raw non-renewable energy by the corresponding amount. The most effective renewable energy to choose is as follows:

40 $$\begin{aligned} j=1,\ldots ,6\underset{}{Max}\left[ (1-\delta )\sum _{i=1}^{4}\left( 1-\epsilon _{r,i,j}\right) \left( {\widetilde{x}}_{r,i,j}^{1}+\frac{\beta _{r}}{1-\beta _{r}}\sum _{i^{\prime }=1}^{4}t_{ii^{\prime }}{\widetilde{x}}_{r,i,j}\right) \right] . \end{aligned}$$

By increasing the efficiency of non-renewables, we can use the less of raw non-renewable energy while keeping the production $[{\mathbf {a}}_{nr}]$. The most effective non-renewable energy is as follows:

41 $$\begin{aligned} \underset{}{j=7,8,9\ Max}\left[ (1-\delta )\sum _{i=1}^{4}\left( 1-\epsilon _{nr,i,j}\right) \left( {\widetilde{x}}_{nr,i,j}^{1}+\frac{\beta _{nr}}{ 1-\beta _{nr}}\sum _{i^{\prime }=1}^{4}t_{ii^{\prime }}{\widetilde{x}} _{nr,i,j}\right) \right] . \end{aligned}$$

**Figure 2:**
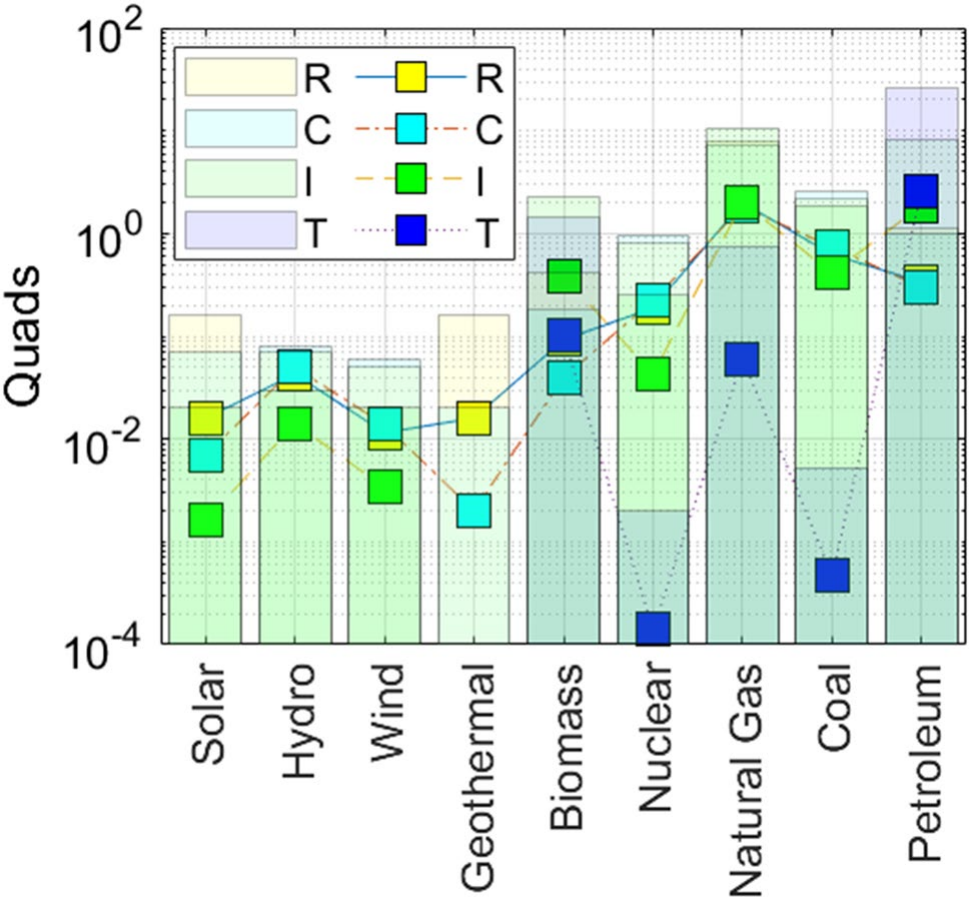
Quad input for each sector. Bar plot for raw energy flow (Eqs. 22–23) and square for efficiency adjusted data (Eqs. 37–39).

The maximum between the above two equations determines the optimal policy response. It is possible that a policy-maker can increase the efficiency of a renewable energy to a greater extent but because of extensive usage, it is better to invest in the improvement of a non-renewable. Figure 2 gives the values for energy flow, $T_{i,j}\cdot x_{r,i,j}$ in Eqs. (22) and (23), using $T=1$. Superimposed on these values, are the (non-aggregate) efficiency adjusted values, ${\widetilde{x}}_{i,j} \otimes \epsilon _{i,j}$. The aggregated values are described in Eqs. (37) – (39).

## Discussion and conclusion

Figure 2 shows the variations in energy flow (shaded bars) for residential, commercial, industrial, and transportation sectors. The efficiency for each of these sectors is also various. Generally, the residential sectors demonstrate an even difference between the level of energy flow and efficiency. The highest efficiency to energy flow values ($x_{i,j}/\epsilon _{i,j}$) includes residential and commercial hydro 0.61 and 0.62, respectively. Energy flows are extracted from Fig. 1; efficiencies from standard production values. The lowest efficiency ratios to energy flow values include the transportation sector using biomass and nuclear fuels, both giving values of 0.07. No efficiencies values are show for solar, hydro, wind, and geothermal for the transportation sector. Also, no efficiency value is given for geothermal in the industrial sector.

Any activity requires energy. Each activity also depends upon other activities. So, characterizing the sustainability of an entire economy requires complex modeling. The present paper has simplified the analysis by reducing the relationships across activities into a matrix format. In the matrix format, we can identify the contribution of each activity in energy consumption in the overall economy. In addition, our approach allows calculating the most effective way to reduce the dependency on non-renewable resources. Future works can focus upon the following topics:

- A subsequent work needs to go beyond matrix analysis to include more general interactions across activities in an economy.
- So far, the analysis does not have actors such as firms or consumers. How the inclusion of such agents affects the analysis needs to be investigated.
- The issue of sustainability involves the impact on future generations. Therefore, the interaction across different generations will be another avenue to pursue.
- The optimal choice of techniques in the context of general sustainability needs to be analyzed as well.
- The social and environmental impacts on energy policies need to be undertaken.

## Data Availability

All data used in this study are available from the corresponding author on request.
